# Do vaccination interventions have effects? A study on how poultry vaccination interventions change smallholder farmer knowledge, attitudes, and practice in villages in Kenya and Tanzania

**DOI:** 10.1007/s11250-018-1679-3

**Published:** 2018-08-17

**Authors:** Johanna F. Lindahl, Jarrah Young, Amanda Wyatt, Mary Young, Robyn Alders, Brigitte Bagnol, Augustino Kibaya, Delia Grace

**Affiliations:** 1 International Livestock Research Institute, PO Box 30709, Nairobi, 00100 Kenya; 2 Swedish University of Agricultural Sciences, Uppsala, Sweden; 3 Uppsala University, Uppsala, Sweden; 4 Kyeema Foundation, GPO Box 3023, Brisbane, Queensland 4001 Australia; 5 International Food Policy Research Institute, 1201 Eye Street, NW, Washington, DC, 20005-3915 USA; 6 School of Life and Environmental Science, Charles Perkins Centre and Marie Bashir Institute, University of Sydney, Sydney, NSW Australia; 7 Department of Anthropology, Witwatersrand University, Johannesburg, South Africa; 8 State Veterinary Services, Dodoma Rural District Council, Dodoma, Tanzania

**Keywords:** Livestock intervention, Chicken, East Africa, Newcastle disease, Backyard farming

## Abstract

Poultry are important for many poor households in developing countries, but there are many constraints to poultry production, including disease. One of the most important diseases of chickens is Newcastle disease (ND). Even though there are effective vaccines against this disease available in most countries, uptake by small-scale poultry keepers is often low. In this study, two areas in Kenya and Tanzania were studied, where some villages had received additional support to get vaccination and other villages had not. In Kenya, 320 households from 10 villages were interviewed, of which half of the villages had active promotion of vaccination through village-based advisors. In Tanzania, 457 households were interviewed, of which 241 came from villages that have had active support through either a project or government extension services. Knowledge about vaccines and the attitudes towards vaccinating against ND was evaluated using mixed multivariable logistic models. Results indicate that in Kenya, the most important determinants for understanding the function of a vaccine were having had support in the village and to have knowledge about ND signs, while in Tanzania gender and previous vaccine use were important in addition to having had support. Attitudes towards vaccination were mainly determined by knowledge, where more knowledge about how vaccines work in general or about ND contributed to more positive attitudes. Among Kenyan farmers that had never used the vaccine before, the amount of birds they lost to disease and predators also influenced attitudes. In conclusion, this study supports the notion that knowledge is a very important component of extension support and that simply making vaccines available may not be sufficient for high levels of uptake.

## Introduction

Poultry are crucial for the livelihoods of rural people especially in developing countries (Alders and Pym 2009). Poor households often keep village poultry, usually chickens that tend to be indigenous birds and require minimal inputs in terms of feed, disease management, and housing. Consumption of village poultry meat and eggs provides an important source of high-quality protein and other micronutrients that are lacking in monotonous diets based around maize which are typical in rural settings in East Africa. In addition, sales of village poultry and eggs are an important source of income for the household and can be used to purchase other foods or pay for school fees or health care. Commonly, village poultry are reared by women in the household and women often retain control over income streams from sales or household’s own consumption (Bagnol 2009). Compared to other livestock, village poultry have low purchase costs, rapid reproduction rates, and the fact that they are easily marketed make them a reliable source of income for the family and affordable even for poorer households to raise.

However, diseases in village poultry can severely hamper productivity and cause financial losses. The impact on livelihoods of poor households can be especially severe. Newcastle disease (ND) is a poultry disease caused by a paramyxovirus, which causes outbreaks in chickens with high mortality (Gallili and Ben-Nathan 1998; Alexander 2001). In areas in which ND is endemic, annual outbreaks of the virus can kill up to 50–100% of the village flock (Alders and Spradbrow 2001). The occurrence of ND in village chickens is often unreported, and thus, estimates of the true incidence are unknown (Alexander et al. 2004). According to official data from 2009, Tanzania had almost 17,000 birds dead from ND, while Kenya only had 128 (AU-IBAR 2009), indicating the extent of underreporting.

Vaccination against ND is the only means of protection available to most parts of the world (Gallili and Ben-Nathan 1998). Vaccination support projects can efficiently reduce bird mortality (Msoffe et al. 2010), and thus have been shown to increase the food security and egg consumption of mothers and children (Knueppel et al. 2010). There are different commercial ND vaccines available today, although these vaccines are often produced for commercial large-scale poultry, with vials containing thousands of doses, which are unaffordable for smallholder farmers (Spradrow and Copland 1996). In addition, the difficulties in maintaining cold chains for vaccines cause further logistic problems, but the development of thermotolerant vaccines, such as I-2, provide solutions for that (Bensink and Spradbrow 1999; Wambura et al. 2000; Shahid Mahmood et al. 2014). Therefore, rural areas often have a delivery system whereby a vaccination vial is distributed to many different households. In order to aid the vaccine dissemination and with the goal to improve productivity in smallholder farming, vaccination programs are often launched by governments or non-governmental organisations; however, programs are often launched in smaller areas and with limited duration. Vaccines against ND are available in both Tanzania and Kenya, and additional support for vaccination of chickens against ND has been provided through projects and non-governmental organisations.

The objective of this analysis was to evaluate the impact of vaccination support on adoption of vaccination and to identify factors for success.

## Material and methods

### Study areas and history of ND vaccine efforts

This study took place in Kenya and Tanzania in East Africa between July and September 2011.

#### Kibwezi District, Kenya

The poultry population in Kenya is estimated to be 31 million, of which 75% are estimated to be indigenous chicken (Zootecnica International 2017). The study was conducted in September 2011 in Kibwezi district, one of the nine districts in Makueni County in Eastern Province. This district was selected because a non-profit organisation, Farm Input Promotions Africa (FIPS-Africa), was promoting vaccination against ND. FIPS-Africa is a not-for-profit company based in Nairobi focused on improving crop productivity of small-scale farmers through the dissemination of appropriate farm inputs and information on their most effective utilisation. At the time, the FIPS-Africa model of ND vaccine delivery relied on district coordinators or village-based advisors (VBAs) to purchase the La Sota vaccine from the local agro-vets. VBAs earned income through provision of the vaccine and actual vaccination of birds in the village. VBAs were financially incentivized through provision of agricultural services and inputs to farmers, such as nurseries for vegetables and trees and sale of other farm inputs. VBAs provided training to farmers on how to use the improved inputs they were selling. The only vaccine provided through this system was the La Sota vaccine, which was the main ND vaccine in use in Kibwezi district at the time of the study.

La Sota is effective, but requires a cold chain. It is recommended to be administered to healthy chickens 2 weeks old or older. The vaccine should be mixed with water shortly before vaccination and then can be added to the chickens’ drinking water or administered via eye-drop or nasal drop. In the village setting, it is common for a 1000-dose vial of the La Sota to be reconstituted at a central point and farmers given portions of the vaccine for their flock. Farmers must rush to their respective homes to deliver the vaccine to their chickens within 2 h of the reconstitution. At home, the water will be provided to thirsty birds and sometimes a syringe is used to drench the chickens. When administered orally, a booster vaccination is required after 2–3 weeks of the first vaccination and re-vaccination performed every 3 months.

#### Chamwino District, Tanzania

In Tanzania, the poultry population is estimated to be 69 million, of which 37 million are estimated to be village poultry (Zootecnica International 2017). The study was conducted between July and August 2011 in Chamwino District, one of the seven districts in Dodoma region, which is currently one of 30 regions in Tanzania. This district was selected because the Southern African ND Control Project (SANDCP) had been active there from 2003 to 2005 in four villages. In addition to SANDCP, extension workers were active in promoting vaccination against ND in a minority of villages, whereas many villages had extension workers who were not actively promoting ND vaccination, and some villages had no extension workers at all (source). SANDCP worked with the Tanzanian government to improve rural food security and the livelihoods of the rural poor through efforts to reduce mortality of smallholder poultry due to ND (Harun et al. 2009). They did this primarily through community education which employed tailored flip charts on ND control, training sessions for community vaccinators and extension officers, and ensuring reliable access to the I-2 ND vaccine (Alders et al. 2002). After the SANDCP project ended, District Councils through the District Agricultural Development Plan (DADP) set aside funds for improving village poultry, including ND control efforts.

The main ND vaccine of use in the study area has been the I-2 ND vaccine. The I-2 ND vaccine is a live, thermotolerant vaccine which was developed by the University of Queensland with funding from the Australian Centre for International Agricultural Research (ACIAR) for local and regional production (Alders and Spradbrow 2001; Young et al. 2012). It is affordable, effective, easy to administer, and does not require a strict cold chain as other ND vaccines (Msami et al. 2006). The I-2 ND vaccine is available in liquid form in Tanzania. It can be administered to chickens of all ages, and in Tanzania, has been recommended to be administered via eye-drop. When refrigeration is available, it should be used to maintain the cold chain. In Chamwino District, where this is rare, storage requirements for liquid I-2 vary depending on whether the vaccine has been opened or not and the ambient temperature. Field research demonstrated that during transportation in the field, wrapping the I-2 ND vaccine in a damp cloth provides evaporative cooling and will maintain the viability of the vaccine for up to 2 days by holding the temperature of the vaccine under 37 °C (Alders and Spradbrow 2001; Chicamisse et al. 2009). Storing the vaccine in direct sunlight or allowing it to reach temperatures above 37 °C for more than 2 days will inactivate the vaccine virus rendering the vaccine ineffective.

Another ND vaccine that is available in the area, mainly in agrovet shops, is La Sota. Experts from the field have suggested that it is likely La Sota would have been used in Mvumi Mission during the time of this study. Electricity is more widely available in this village compared to villages in other parts of the district, so proper storage of the vaccine is possible.

### Sampling and data collection

For this study, a sampling frame of villages in Kibwezi District was obtained and five villages that had benefited from the vaccination support by FIPS were randomly selected, as well as five villages that had not benefited. In each village, 32 households were randomly selected and interviewed by trained enumerators during September 2011. This sample size would allow us to find a difference of 15% between the groups with a power of 0.8 (power two proportions 0.5, test(chi^2^) power(0.8) *n*(320)).

In Chamwino District, State Veterinary Services personnel within the Dodoma Rural District Council provided a list of villages broken down into four pre-determined strata: villages which had participated in SANDCP from 2003 to 2005 (“project villages”), villages with agriculture extension services active in promoting ND vaccination, villages with agriculture extension services that were not active in promoting ND vaccination, and villages without any agriculture extension officers. Two villages from each of the four categories (SANDCP, active extension, inactive extension, no extension) were randomly selected, and the two first categories were classed as villages with support. A list of households was compiled by the sub-village chiefs followed by verification visits by the village extension officers, and necessary corrections were made before arriving at a final census. During July and August 2011, 48 to 72 households (proportional to the number of households in the villages) were randomly selected and interviewed by trained enumerators.

Household questionnaires were developed in English and translated into Kiswahili. Face-to-face interviews were conducted with one household member regardless of gender or position in the household, although enumerators were asked to aim for an even balance of male and female respondents in each site. In both sites, data was collected on participant’s gender and age, gender of the household head, animals kept in the households, which animals were considered most important, and if poultry was considered important, the reasons for this. Further, data was collected on the losses of poultry during the previous year, if vaccination had ever been done, if so how long this had been, and who decided on the vaccination, who performed it, and the price.

Knowledge and attitudes about vaccines, ND, and the ND vaccine were assessed through different questions. For example, a person was judged to know what a vaccine is if they said a vaccine prevents a specific disease but not if they said it prevented all diseases or cured diseases. Knowledge about ND was judged based on how many answers were correct of seven questions which required identifying five clinical signs of ND, and also rejecting two clinical signs of other diseases not associated with ND. Knowledge about ND vaccines was based on seven questions about vaccine distribution; in Tanzania, a question on how long the time was between the vaccine administration and the time when the vaccine was protective was added, and thus, knowledge on ND vaccines was based on eight questions.

### Data analyses

Survey data were captured on paper forms and entered into Excel. Statistical analyses were performed using STATA 11.2 (STATA corp, Texas, USA). Univariable analyses between categorical variables were performed using chi-square or Fisher’s exact test where applicable. Univariable associations between count and continuous data were tested using *t* test, and between continuous variables using regression. Based on causal diagrams, all independent variables believed to affect three variables—use of ND vaccine, knowledge of vaccine in general, and attitudes towards ND vaccination—were analysed using multivariable binomial regression with the xtmelogit procedure, with village as a random effect. When attitudes were analysed, the binomial regression was based on five groups of binomial data. Backwards elimination was done manually, keeping variables with *p* values under 0.05 or confounding significant variables by more than 20%. Interactions were tested between the remaining variables and kept if significant at a 0.05 level. Models were evaluated based on likelihood ratio tests, residual analyses, and predictive capacity.

In the multivariable models for whether a household had used ND vaccine or not, associations were tested with the total number of chickens in the household, whether chickens were considered the most important animals, the gender of the household head, the number of chickens lost to ND and to predators respectively during the last year, the knowledge of ND clinical signs, and whether or not the village had had any vaccination support. A separate model was also built to see associations between these factors only within the households in supported villages. In the models for general knowledge of what a vaccination does, the same independent variables were used, in addition to whether or not the household had previously used ND vaccine.

Attitude scores were analysed for all respondents and stratified by having ever used the ND vaccine. Thus, attitudes could be interpreted based on those with experience using the ND vaccine and based on attitudes and beliefs, not experience. In the analysis of attitudes among those who had used ND vaccine in Kenya, respondents who were not in support villages (*n* = 2) were excluded for model fit. These models were tested for associations with the numbers of chickens in total in the household, the numbers of chickens lost to ND and to predators last year, the gender of the household head, the knowledge of ND symptoms and ND vaccine, general knowledge of vaccines, if chickens were considered the most important animals and where applicable, also previous use of ND vaccine and if the village had received support.

In the models for attitude towards vaccine use, associations were tested with the total number of chickens in the household, whether chickens were considered the most important animals, the gender of the household head, the number of chickens lost to ND and to predators respectively during the last year, the knowledge of ND clinical signs and about ND vaccine, the general knowledge of what a vaccine does and, where applicable, whether or not the village had had any vaccination support, and if the household had used ND vaccines previously.

## Results

In total, 316 households in Kenya and 456 households in Tanzania agreed to participate in the household survey. In Kenya and Tanzania, 50.6% and 52.7% of the interviewed households respectively were in villages that had received additional vaccination support. Where vaccination support had been given, 59.1% of households overall had used vaccines against ND, which was significantly (*p* < 0.001) more than the 16.9% of households who had used the ND vaccine in areas with no additional support. Only two households in unsupported villages in Kenya had ever used ND vaccines. In the multivariable analysis of factors associated with ever having used ND vaccines in Kenya, only support to the village had a significant effect (*p* < 0.001), with an increased odds ratio (OR) of 147 (95% confidence interval (CI) 35.1–615.8). The separate model for households only in supported villages did not find any significant associations. In Tanzania, the households in supported villages also had significantly higher odds of ever having used the vaccine (OR 4.25, 95% CI 1.54–11.8, *p* = 0.005) but the odds also increased with the numbers of chickens owned (OR 1.03, 95% CI 1.01–10.5, *p* < 0.001).

**Fig. 1:**
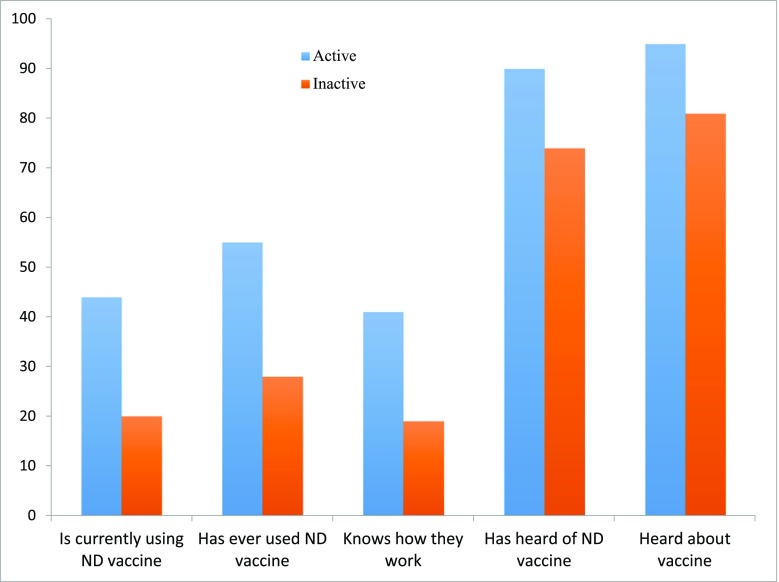
The percentage of households knowing about Newcastle disease (ND) vaccine in Kenya and Tanzania, segregated on if the village had had active vaccination support or not

In the majority of households, 72.8% in Kenya and 78.6% in Tanzania, the household heads were male. In 83.7% of households, the household head made the decision of whether or not to vaccinate the poultry, although there were significant differences in decision-making patterns (*p* = 0.001) between Kenya (89.1%) and Tanzania (73.8%), but no significant difference between female and male-headed households. In Kenya, a significantly (*p* < 0.001) higher proportion of male-headed households (94/230) than female-headed (13/86) had used ND vaccine in univariable analysis. In both Kenya and Tanzania, the use was higher in supported villages (Table 1). For all knowledge questions about the vaccine, a higher proportion in villages with vaccination support knew about it (Fig. 1).

**Table 1:** Use of Newcastle disease vaccine in supported and non-supported villages in Kenya and Tanzania

		Active support village	Inactive village	Chi *p* value
Kenya	316	105/160 (65.6%)	2/156 (1.3%)	< 0.001
Tanzania	457	132/241 (54.8%)	61/216 (28.2%)	< 0.001
Both	773	237/401 (59.1%)	63/372 (16.9%)	< 0.001

Farmers in villages which had received support were more correct in their knowledge of what a vaccine does than other farmers (*p* < 0.001), with 33.1% of respondents in non-supported villages having knowledge, compared to 59.6% in supported villages. The results of the multivariable analyses are shown in Table 2.

**Table 2:** Factors influencing the knowledge of Newcastle disease (ND) vaccines among poultry farmers in Kenya and Tanzania

Know what vaccines do		OR	95% CI	*p*
Kenya	Knowledge of ND clinical signs	1.79	1.30–2.47	< 0.001
	Vaccination support in village	3.39	1.02–11.26	0.047
Tanzania	Ever used ND vaccine	3.03	1.91–4.80	< 0.001
	Vaccination support in village	3.17	1.31–7.62	0.01
	Gender of the household head	2.67	1.54–4.60	< 0.001
	Chickens lost to ND last year	1.01	1.00–1.03	0.04

There were also significant differences in farmers’ attitudes towards vaccination depending on if their village had received support or not. In villages that had received support, farmers were more prone to report that vaccine was affordable (*p* < 0.001), that it was not difficult to administer (*p* < 0.001), and that it was effective (*p* < 0.001), but there was no significant difference in the number of farmers that considered the birds difficult to catch. The results of the multivariable analyses are shown in Table 3.

**Table 3:** Attitudes towards Newcastle disease (ND) vaccine among poultry farmers in Kenya and Tanzania, analysed separately for farmers that never had used the vaccine and farmers that had used it previously

Attitudes towards ND vaccine		OR	95% CI	*p*
Kenya				
Never used ND vaccine	Knowledge of ND clinical signs	1.29	1.09–1.53	0.003
	Vaccination support in village	0.52	0.28–0.95	0.035
	Know what a vaccine does	1.83	1.26–2.65	0.001
Used ND vaccine	Know what a vaccine does	5.13	2.42–10.87	< 0.001
Tanzania				
Never used ND vaccine	Knowledge of ND clinical signs	1.48	1.22–1.79	< 0.001
	Knowledge of ND vaccine	2.08	1.54–2.81	< 0.001
	Interaction between knowledge of ND vaccine and ND	0.88	0.82–0.95	0.001
	Chickens lost to predators last year	0.98	0.97–1.00	0.013
	Chickens lost to ND last year	1.01	1.00–1.02	0.045
Used ND vaccine	Knowledge of ND vaccine	1.22	1.11–1.35	< 0.001
	Know what a vaccine does	1.59	1.17–2.17	0.003
	Total chickens in the household	1.01	1.00–1.02	0.035

In Kenya, 72.9% (78/107) of the households that had ever used ND vaccine continued to use it after the first month. In Tanzania, only 45.1% (87/193) continued to use, but there were significant differences (*p* < 0.001) between households in supported villages where 58.3% of households continues, compared to 16.4% in non-supported villages.

In Tanzania, farmers that had not received support reported an average loss of 17.5 (standard deviation 1.1) birds due to disease the previous year, while those from villages that had received support only 10.3 birds lost to disease (SD 0.66) (*p* < 0.001). Similarly, in Kenya, there was also a significant (*p* < 0.036) difference between farmers from villages with and without (4.6 birds (SD 0.54) and 6.7 birds (SD 0.84)).

## Discussion

In this study, the effects of current and previous ND vaccination initiatives on the use of vaccines and the attitudes towards vaccination are investigated. The use of vaccine was highly associated with previous vaccination support. In Kenya, only two households from villages without support had ever vaccinated their chickens. Extension services have been shown to be an important factor for predicting the adoption of interventions by poultry farmers (Ochieng et al. 2012), and in this study, the presence or absence of support was included as the main predictor even though the mode varied between villages.

Understanding how a vaccine works was associated with village support in both countries. In Kenya, it was associated with knowledge of ND clinical signs, and in Tanzania with the number of chickens lost to ND. Both of these could be indicators of how important ND has been to the household and indicate that households that have been more exposed to ND are more knowledgeable about vaccines or more prone to seek out information or learn when information is offered. Knowledge that a vaccine only protects against specific disease is important for attitudes and continued vaccine use, since high expectations that the vaccine will protect against all diseases are likely to make farmers disappointed and more negative towards vaccines (Alders et al. 2003).

In Kenya, having general knowledge about vaccines was associated with more positive attitudes to ND vaccine, both among the households that had tried the vaccine and those that had not. Interestingly, households in villages with vaccination support that had never used the vaccine were more negative towards the vaccine. One explanation for this could be that mainly the people with prior wholly negative attitudes towards vaccination, possibly due to previous negative experiences, were the ones who choose not to vaccinate when support was offered. In Tanzania, increased knowledge of vaccines and ND was associated with more positive attitudes towards vaccinating. In households that had never tried the vaccine, it seems as if losing many chickens to ND made farmers more positive towards vaccination, whereas losing chickens to predators made them less positive. A likely explanation for these differences could be that farmers perceive it to be a waste to vaccinate birds if there is a high likelihood that the animals will be taken by predators.

It was not possible to determine which type of vaccine had been used by farmers, and therefore, it is impossible to assess if any differences in attitudes are due to different vaccines being used. Keeping of a cold chain as is required for the La Sota vaccine can be a challenge in remote villages (Foster et al. 1999), and high temperatures may reduce the efficacy of the vaccine, which could make farmers perceive that the vaccine is not efficient. The I-2 strain was especially selected for its thermotolerant capacities to be used in developing countries (Bensink and Spradbrow 1999). The strain has been shown to be equivalent to La Sota in laboratory conditions, give a good immunity, and work well in village poultry (Dinh et al. 1998; Henning et al. 2009).

There was no direct assessment in this study of how many households actually vaccinated chickens the recommended three times a year; however, the data suggested only a minority of farmers were vaccinating at this frequency. This is in accordance with a previous study in Tanzania (Knueppel et al. 2010) that showed that only a small proportion of households (13–20%) vaccinated the required amount of times, even if the vaccine was provided free by a project or provided at a low cost.

The estimates on the number of chickens lost to ND in the households were based on the farmers’ perception. ND can be per-acute and fatal without preceding clinical signs, and signs can also be protean, and not pathognomonic (Alexander et al. 2004). There can therefore be some inaccuracies in these numbers, but since the most important for the attitudes of the farmers is likely to be the perception, rather than the actual number, it was still used in the analyses.

A study in Tanzania found that more than 80% of the poultry could be seropositive to ND (Yongolo et al. 1996), but considering that meta-analysis estimated seroprevalences to be less than 1% (Miguel et al. 2013), it is unlikely that the former findings are representative.

An interesting finding was that vaccination decisions in this study were mainly made by men. Other research has found that poultry are particularly important for women (Bagnol 2009) and that gender is an important factor for adoption of extension services, including vaccination (Ochieng et al. 2012). Information about vaccination in Kenya and Tanzania may therefore need to target both men and women to reach the desired effect.

Although two different countries were included in the analyses, where different vaccination support had been given, an overall finding shows that having been present in a village with a vaccination initiative not only was associated with increased vaccine usage, but also with more positive attitudes towards the vaccine. This may be attributed to increased knowledge about the effects of vaccines in general or to the positive effects caused by increased vaccination to poultry production.

## Conclusion

Poultry keeping is an important source of nutrition and income for poor families in many low and middle-income countries. Infectious diseases pose a threat to this, and a disease such as ND can extinguish the entire flock of a family. This study shows that interventions where households are given support for vaccinating their animals, even during a limited time, can significantly change the knowledge of and attitudes towards the vaccine. Most importantly, it shows that one important factor for the attitudes towards vaccination is knowledge, both generally of the principle of a vaccine and specifically how vaccination should be done. Improving knowledge can thus contribute to creating increasing demands for, and uptake of, vaccines, and thus significantly decreasing mortality. Therefore, information must be an important part of vaccination projects in order for vaccine use to be sustainable.
